# Supplementary material for: Systematic Comparison of Hospital-Wide Standard and Model-Based Therapeutic Drug Monitoring of Vancomycin in Adults
Source: Pharmaceutics. 2022 Jul 13;14(7):1459. doi: 10.3390/pharmaceutics14071459 (PMC9320266; doi:10.3390/pharmaceutics14071459)
Supplement: Supplementary file 1 [file pharmaceutics-14-01459-s001.zip › pharmaceutics-1795982-supplementary.pdf]

pharmaceutics

*Supplementary Materials*

# Systematic comparison of hospital-wide standard and model-based therapeutic drug monitoring of vancomycin in adults

**Heleen Gastmans [1,#], Erwin Dreesen [2#], Sebastian G. Wicha [3], Nada Dia [2], Ellen Spreuwers [1], Annabel Dompas [4], Karel Allegaert [2,5,6], Stefanie Desmet [7,8], Katrien Lagrou [7,8], Willy E. Peetermans [9,10], Yves Debaveye [11], Isabel Spriet [1,2,†], Matthias Gijsen [1,2,†]**

pharmaceutics MDPI

## SUPPLEMENTARY MATERIALS

**Supplementary Table S1.** Acceptable performance and classification accuracy across the five investigated model-based approaches. Acceptable performance is defined as a bias ≤ ±2 mg/L for concentrations below 20 mg/L or a rBias ≤ ±10% for concentrations of 20 mg/L and higher. Classification accuracy is defined as no change in exposure category (i.e., subtherapeutic, supratherapeutic or therapeutic) according to the predicted vancomycin concentration compared to the observed concentration.

| **Model-based approach** | Acceptable performance n (%) | Classification accuracy n (%) |
|---|---|---|
| **Goti** | 118 (38.3) | 147 (47.7) |
| **$MSA_{OFV}$** | 129 (41.9) | 171 (55.5) |
| **$MSA_{SSE}$** | 143 (46.4) | 166 (53.9) |
| **$MAA_{OFV}$** | 117 (38) | 148 (48.1) |
| **$MAA_{SSE}$** | 140 (45.5) | 166 (53.9) |

MAA: model averaging algorithm; MSA: model-selection algorithm; OFV: objective function value; SSE: squared prediction errors.

**Supplementary Table S2.** Relative bias (rBias) with 95% confidence interval (CI) and relative root mean squared error (rRMSE) of the five model-based approaches investigated in this study in the total population and in the subpopulations.

| | **Total** | | **Non-ICU** | | **ICU** | | **Intermittent infusion** | | **Continuous infusion** | |
|---|---|---|---|---|---|---|---|---|---|---|
| **Model-based approach** | rBias (%) [95% CI] | rRMSE (%) | rBias (%) [95% CI] | rRMSE (%) | rBias (%) [95% CI] | rRMSE (%) | rBias (%) [95% CI] | rRMSE (%) | rBias (%) [95% CI] | rRMSE (%) |
| **Goti** | 3,18 [-1,42;7,77] | 28,1 | 4,35 [-0,25;8,95] | 28,1 | 1,39 [-3,21;5,99] | 28,09 | 7,23 [2,63;11,83] | 27,45 | -3,35 [-7,95;1,25] | 29,11 |
| **$MSA_{OFV}$** | -5,36 [-9,83;-0,9] | 27,14 | -3,88 [-8,35;0,58] | 27 | -7,62 [-12,09;-3,16] | 27,35 | -2,38 [-6,84;2,09] | 22,94 | -10,18 [-14,64;-5,71] | 32,78 |
| **$MSA_{SSE}$** | -2,54 [-7,12;2,04] | 26,05 | -1,16 [-5,74;3,42] | 26,41 | -4,65 [-9,23;-0,07] | 25,5 | -1,16 [-5,74;3,42] | 21,46 | -4,77 [-9,35;-0,18] | 32,09 |
| **$MAA_{OFV}$** | -4,35 [-8,71;0,01] | 26,92 | -3,21 [-7,57;1,16] | 27,09 | -6,1 [-10,46;-1,74] | 26,67 | -1,36 [-5,72;3] | 23,26 | -9,17 [-13,53;-4,81] | 31,95 |
| **$MAA_{SSE}$** | -2,7 [-6,92;1,53] | 24,8 | -1,33 [-5,55;2,89] | 24,94 | -4,78 [-9,01;-0,56] | 24,58 | -1,52 [-5,75;2,7] | 20,68 | -4,59 [-8,81;-0,36] | 30,28 |

CI: confidence interval; ICU: intensive care unit; MAA: model averaging algorithm; MSA: model-selection algorithm; OFV: objective function value; rBias: relative bias; rRMSE: relative root mean squared error; SSE: squared prediction errors.

**Supplementary Table S3.** Clinically relevant differences between model-predicted and prescribed vancomycin doses according to the exposure at the time of the second vancomycin concentration of each pair.

[a] < 12.5 mg/L (intermittent), < 20 mg/L (continuous); [b] > 17.5 mg/L (intermittent), > 25 mg/L (continuous); [c] 12.5-17.5 mg/L (intermittent), 20-25 mg/L (continuous);

| **Difference in model-predicted dose compared to prescribed dose** | **Subtherapeutic[a] exposure at second concentration** | **Supratherapeutic[b] exposure at second concentration** | **Therapeutic[c] exposure at second concentration** |
|---|---|---|---|
| **Dose increase, n (%)** ***≥ +125 mg*** | 32 (48.5) | 23 (24.2) | 53 (36.1) |
| **Dose reduction, n (%)** ***≤ −125 mg*** | 14 (21.2) | 38 (40) | 24 (16.3) |
| **Dose equal, n (%)** ***< +125 mg & > −125 mg*** | 20 (30.3) | 34 (35.8) | 70 (47.6) |
| **Total** | 66 | 95 | 147 |

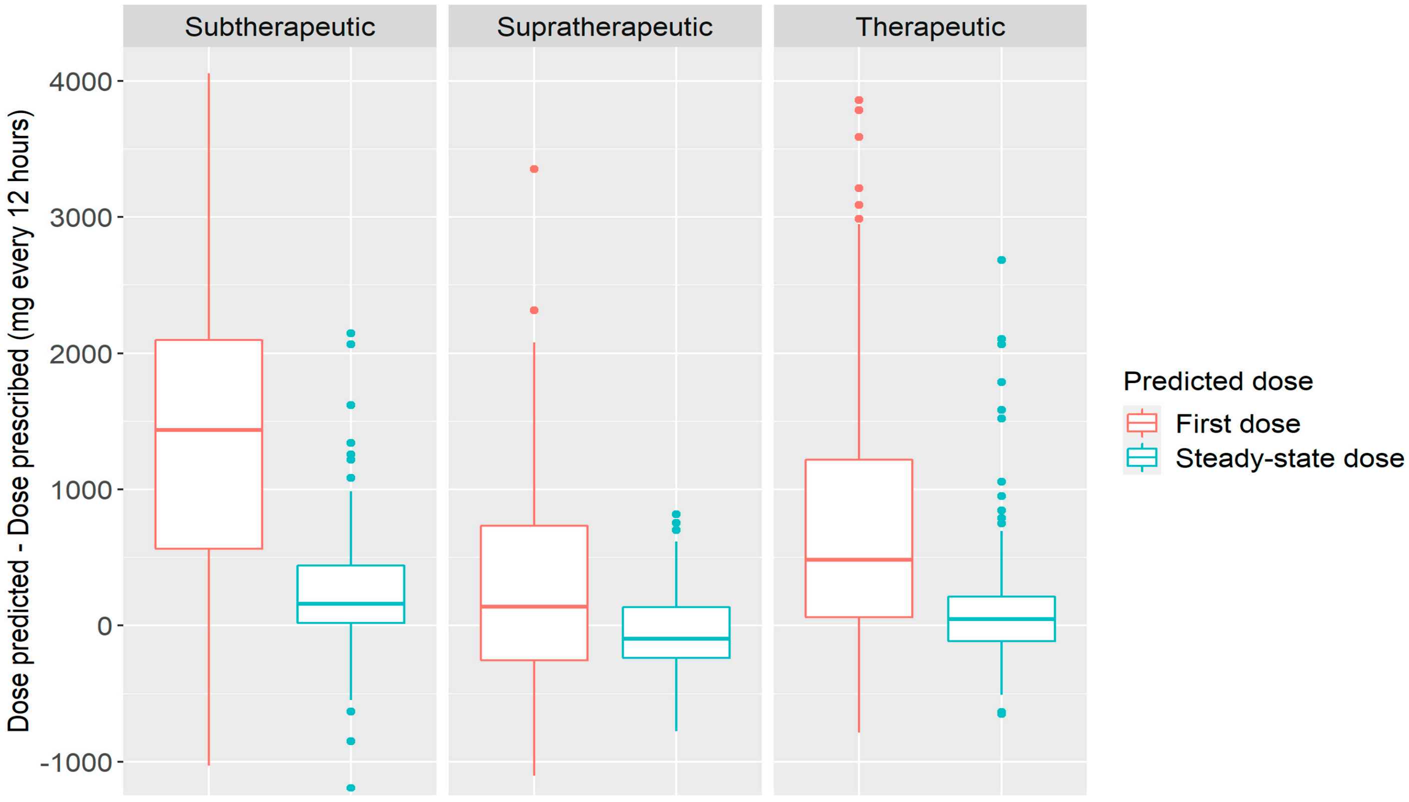


| | Subtherapeutic | | Supratherapeutic | | Therapeutic | |
|---|---|---|---|---|---|---|
| **Median difference [IQR], mg every 12h** | 1436 [564;2096] | 160 [19;442] | 137 [-256;735] | -98 [-237;135] | 483 [61;1218] | 47 [-116;213] |

**Supplementary Figure S1.** Boxplots of the difference between vancomycin doses predicted by the $MSA_{OFV}$ approach and prescribed vancomycin doses, including the median [interquartile range] difference. Doses are normalized to a twice daily dosing regimen, i.e., doses are expressed as dose q12h. Differences are shown in three groups depending on the exposure at the time of the second vancomycin concentration of each pair (i.e., subtherapeutic, supratherapeutic or therapeutic). Vancomycin doses were predicted based on the first vancomycin concentration of each pair of concentrations. The red and blue boxplots represent the dose differences based on the first and second dose predicted to reach therapeutic exposure, respectively. Therapeutic exposure was defined as concentrations between 12.5-17.5 mg/L or between 20-25 mg/L for intermittent or continuous infusion, respectively.
